# Dynamic Task Allocation in Multi-Hop Multimedia Wireless Sensor Networks with Low Mobility

**DOI:** 10.3390/s131013998

**Published:** 2013-10-16

**Authors:** Yichao Jin, Serdar Vural, Alexander Gluhak, Klaus Moessner

**Affiliations:** Centre for Communication Systems Research (CCSR), University of Surrey, Guildford GU2 7XH, UK; E-Mails: s.vural@surrey.ac.uk (S.V.); a.gluhak@surrey.ac.uk (A.G.); k.moessner@surrey.ac.uk (K.M.)

**Keywords:** multi-hop multimedia wireless sensor network, task allocation, low mobility, genetic algorithm

## Abstract

This paper presents a task allocation-oriented framework to enable efficient in-network processing and cost-effective multi-hop resource sharing for dynamic multi-hop multimedia wireless sensor networks with low node mobility, e.g., pedestrian speeds. The proposed system incorporates a fast task reallocation algorithm to quickly recover from possible network service disruptions, such as node or link failures. An evolutional self-learning mechanism based on a genetic algorithm continuously adapts the system parameters in order to meet the desired application delay requirements, while also achieving a sufficiently long network lifetime. Since the algorithm runtime incurs considerable time delay while updating task assignments, we introduce an adaptive window size to limit the delay periods and ensure an up-to-date solution based on node mobility patterns and device processing capabilities. To the best of our knowledge, this is the first study that yields multi-objective task allocation in a mobile multi-hop wireless environment under dynamic conditions. Simulations are performed in various settings, and the results show considerable performance improvement in extending network lifetime compared to heuristic mechanisms. Furthermore, the proposed framework provides noticeable reduction in the frequency of missing application deadlines.

## 1. Introduction

The growing need to support high performance applications in multi-hop multimedia wireless sensor networks (MWSNs) [1] while coping with limited node capabilities [2] highlights the necessity of resource sharing and node collaboration [3-5]. For example, in a surveillance sensor network consisting of wireless camera nodes [6,7], real-time computation of large amounts of visual data and performing complex image processing-based algorithms in resource constrained sensor nodes imposes news challenges for MWSN design. On the other hand, transmitting all the raw image data via multi-hop wireless communication to a remote gateway or to the cloud and retrieving the computation results is very costly in terms of energy consumption, as well as large time delays on the order of seconds. Hence, multimedia in-network processing [8] could be one solution to this problem, which divides a computationally demanding program into smaller tasks and, then, intelligently assigns them to a set of nodes in order to efficiently use available network resources. However, additional costs may occur, due to the multi-hop wireless communication that is required to exchange information among individual tasks. Hence, task allocation algorithms have to consider the trade-off between processing and communication costs.

Network dynamicity causes additional complexity in a task allocation system. For example, in an earthquake relief use case, multiple collaborative agents equipped with cameras are dispatched to the emergency scenes to carry out time-critical missions, such as search and rescue [9], and form a dynamic MWSN. However, when a critical agent/node leaves the network, due to communication interruption or physical node failure, serious consequences, such as network service disruption, can occur. In such cases, control messages are exchanged among nodes in order to isolate the faulty ones and detect the affected tasks that need to be immediately reallocated to suitable nodes. Furthermore, stochastic movements of a patrolling agent might affect its own communication or cause interference on its neighbours. This implies that the effectiveness of a fixed task allocation solution may degrade and eventually become invalid if there is no update for the solution based on the latest network conditions. The simplest reaction is to regard each change in the network topology as the arrival of a new task allocation problem that has to be solved from scratch by re-running the allocation algorithm. Nevertheless, due to the complexity of MWSNs, assessments of finding a qualified solution are often computationally time consuming, which has a direct effect on the quality of the computed solution for time-critical applications.

### 1.1. Motivation

In this paper, the problem of dynamic task allocation and scheduling in MWSNs is considered. Algorithm complexity and the corresponding runtime to produce and update solutions are explicitly taken into account. Existing sophisticated heuristic algorithms [10,11] are not suitable for dynamic network conditions, due to their algorithm complexity. In contrast, simple and fast algorithms run the risk of providing only low quality solutions. A genetic algorithm (GA) is a possible alternative to these heuristic approaches, as a GA is typically designed for multi-variable settings and is shown to be efficient in solving task allocation and scheduling problems [12,13]. However, GAs are time consuming and, hence, cannot be directly applied to networks with dynamically changing conditions or topologies. On the other hand, the execution of a GA can be divided into several sequential stages [14], each of which requires less resource and executes more quickly. Seeing this fact, the conjecture is that it is possible to provide an intermediate result of a GA, which is sub-optimal, as a fair solution that suits the latest conditions in a dynamic network. Furthermore, the quality of the provided solutions can be improved over time by progressively enhancing the pool of solutions, called the GA population, using an efficient and fast heuristic that makes corrections based on network changes. Therefore, in this paper, the objective is to develop a framework that is a combination of an evolutional GA and a heuristic to strike the balance between algorithm execution time and adaptability to network dynamics.

### 1.2. Main Contribution

In this paper, the Dynamic Task Allocation and Scheduling (DTAS) framework is presented. DTAS aims at minimizing the frequency of instances when an application misses an arbitrarily set deadline (*deadline miss ratio*), while also extending network lifetime by balancing node energy consumption levels. To the best of our knowledge, this is the first study that provides multi-objective task allocation in complex and dynamic multi-hop network environments.

DTAS can be summarized as follows: First, a heuristic minimum hop count algorithm is designed to guide the initial solution creation, which can effectively reduce problem complexity. Second, a self-learning process (SLP) based on a GA is applied, which continuously evolves a set of solutions, so that multiple design objectives can be met. Intermediate results of SLP can be provided as temporary sub-optimal solutions to cope with changing network conditions. The fitness function in SLP initially favours meeting the deadline requirement and, then, gradually leans towards a balanced solution between task execution time and network lifetime. An adaptive window is proposed to keep the GA execution time under control, such that the final solution is up-to-date with the most recent network conditions. Finally, to deal with sudden node or link failure events and to update the solutions in SLP, a Fast Task Recovery Algorithm (FTRA) is designed to quickly reallocate faulty task assignments.

### 1.3. Related Work

The task allocation problem in parallel and distributed systems has been extensively studied in both wired and wireless networks. Existing solutions are based on multi-objective optimization approaches considering: minimizing task completion time [15,16], reducing energy consumption [11,17,18], load balancing to achieve an equalized node lifetime [19,20] and maximizing service reliability [21]. In wired networks, since nodes are often connected with dedicated and high quality links, communication costs and delays are often considered to be negligible. However, the situation in an MWSN is quite different, and solutions like [17,19] and [22] consider both processing costs and wireless communication costs. Integer Linear Programming is adapted in these approaches to solve the problem of energy-efficient task mapping and scheduling with deadline constraints. Nevertheless, these algorithms are based on a single-hop topology, and the time to compute the optimized solution is not added to the overall cost, which hinders their applicability in large-scale networks.

Heuristic approaches are deterministic and non-backtracking, since task allocation decisions cannot be changed, even if the decisions are found to be inappropriate at a later stage of the algorithm execution [11]. Therefore, solutions are likely to be prone to errors, especially in dynamic MWSNs. To overcome this issue and provide optimal solutions, genetic algorithm (GA)-based multi-hop task allocation schemes are proposed in [13,20]. Nevertheless, such schemes can only work on static network conditions and have high time-complexity; hence, they can only provide off-line optimization. In contrast, recent work in [23,24] consider dynamic task allocation in wireless environments, yet only single-hop communication is taken into account. Furthermore, most of these studies [11,13] assume a relatively powerful machine that is capable of running an optimization algorithm whilst meeting task deadlines. However, such an assumption implies extra hardware cost, hence significantly limiting the applicability of these algorithms in embedded systems.

The rest of the paper is organized as follows. In Section 2, the models and assumptions are presented, followed by the addressed research problem. Section 3 covers the technologies developed for task allocation in MWSNs. Then, the proposed DTAS framework is presented in Section 4, and the effectiveness of the design is illustrated in Section 5. Finally, Section 6 concludes the paper.

## 2. Preliminaries

### 2.1. System Models

A Directed Acyclic Graph (DAG) *G* = (*T*, *E*) is used to model an application [11,19]. Each vertex in the DAG represents a task *T_i_* ∈ *T* that is connected to other vertices by directed edges. Each task, *T_i_*, has a workload, *p_i_*, representing the processing requirement in terms of the number of CPU clock cycles to execute the task. The weight on each edge, *e_ij_*, stands for the amount of data transmitted from *T_i_* to *T_j_*. A direct edge (*e_ij_* ∈ *E*) shows the precedence relations among tasks, *i.e. T_i_* should be completed before *T_j_*. Therefore, a DAG has a topological task execution order, which we term the task scheduling sequence (TSS). Furthermore, an application can iteratively execute the DAG. A *round* is defined as the time period of a DAG execution cycle.

The network topology consists of a total number of *M* heterogeneous nodes *V* = {*v*_1_, *v*_2_, ⋯, *v_M_*} that are randomly deployed in the network. For simplicity, transmission power control is not enabled. Hence, all nodes have a fixed communication range, and they are connected via multi-hop links. Nodes are battery powered, and each node has a finite energy supply that is not refilled. Heterogeneous initial battery energy and processing speeds are considered. However, it is assumed that the gateway is much more powerful and easier to be maintained (e.g., recharge) compared to the remotely distributed nodes. A non-preemptive scheduling policy is adopted, so that only one job can be processed at each node at a time. It is assumed that nodes are synchronized and that the wireless channel condition is stable. Furthermore, in order to perform scheduled multi-hop communication, a bandwidth reservation mechanism is used, such as a TDMA (time division multiple access) based MAC (media access control) protocol [25,26]. Unless specified otherwise, each task is executable at every node.

The network dynamics considered in this study has the following properties:

1. As an example application, surveillance networks with movable agents equipped with wireless cameras are considered, in which each agent has a probability, *p_move_*, to move at a pointed or random direction with a speed of *ν_move_* in each round. At present, a pedestrian moving speed is assumed for *ν_move_* (between [0.91, 1.22] *m*/*s*).
2. Each node has an exponential distribution of failure probability *p_f_*(*t*) = 1 − *e*^−λ^*^t^*, where λ is the average node failure rate in the time interval [0, *t*] [21].

Communication links in the network may change over time because of these random changes. However, each node has regular gossip message exchanges with its neighbours and periodically reports its own *neighbour list* to a central network controller (the gateway). Based on the collected information, the network link topology, 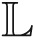, is updated periodically. A dedicated control channel is used for these message exchanges, whose energy consumption is included in the total cost calculation.

### 2.2. Definitions

The terms used in the rest of the paper are as follows:

1. *Network lifetime (NL):* The time period until the first node fails due to energy depletion.
2. *Schedule length (SL):* The execution time of a DAG.

### 2.3. Problem Definition

The problem that this paper addresses is two-fold. First, an optimized task allocation solution, *s*, is to be found with the objective of maximizing the network lifetime, *NL*, under the required time-delay constraints. To achieve this, the total schedule length, *SL*, must meet the deadline, *t_deadline_*. Hence, the objective function can be formulated as follows:

(1) $$\begin{array}{l} \max\left\{NL(s),\;s\in \text{total search space}\right\}\\ \text{subject to}:\;SL(s)\leq t_{\text{deadline}} \end{array}$$

Secondly, the chosen solution, *s*, should be able to update itself, such that it can adapt to network dynamics. However, this is a challenging task because of the following reasons:

1. *Node mobility and node failure events:* The optimized task allocation solution may become invalid when such events occur. Re-assigning the affected tasks can only serve as a temporary solution, as re-optimization is required according to emerging network conditions. Nevertheless, due to the problem complexity, a complete re-run of the algorithm is costly.
2. *Algorithm runtime and complexity:* The proposed task allocation algorithm runs at the gateway node, and its algorithm runtime is denoted by *K*. In static networks, a high-cost algorithm can work perfectly well as an off-line solution. On the other hand, algorithm runtime is critical in dynamic environments. Since optimization parameters have to be quickly modified in order to adapt to changing conditions, optimization procedures that require a large value of *K* to complete are likely to produce outdated solutions in dynamic environments.

**Figure 1.**
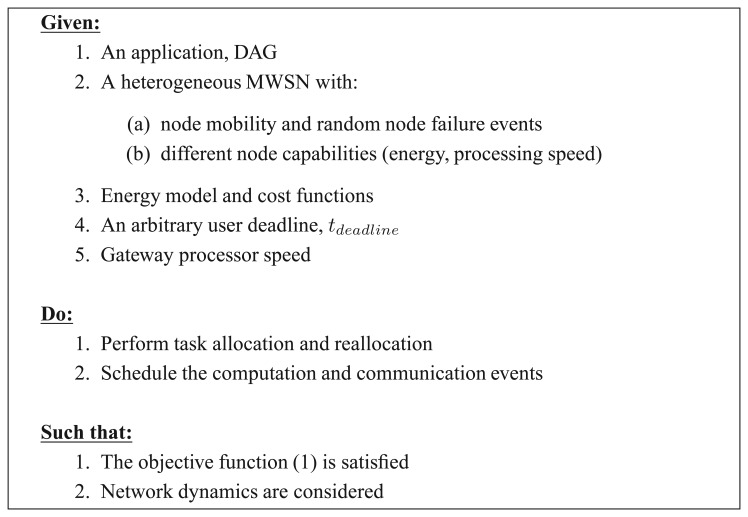
Design objectives.

The main design objectives can be summarised as shown in Figure 1.

In the following, first, how static task allocation and scheduling is performed in MWSNs is explained. Then, Section 4 presents how the *dynamic* allocation problem can be solved using our Dynamic Task Allocation and Scheduling (DTAS) framework.

## 3. Task Allocation and Scheduling in MWSNs

In a DAG, *G*, a task pair (*T_i_*, *T_j_*) connected by a directed edge, *e_ij_*, could be allocated to nodes that are several hops away from each other in the network. Therefore, multi-hop communication costs must be included in the task allocation solution structure. Furthermore, task scheduling in an MWSN needs to take into account particular issues, like parallel processing among independent nodes, possible simultaneous communications and multi-cast transmissions. To tackle these issues, in our previous work [13], we developed a task allocation model and a multi-hop scheduling mechanism for *static* MWSNs. Since the proposed DTAS presented in Section 4 is based on this model, we briefly describe it in this section.

### 3.1. Multi-Hop Extension of Task Allocation

For a solution, *s*, to be evaluated in a multi-processor environment, first, an encoding process transforms *s* into individual tasks that can be independently processed. Then, an initial mapping of these tasks to network nodes is performed, which is modeled by a three-by-*δ* matrix, *C*, called the *chromosome*, where *δ* is the total number of the tasks in the DAG.

**Figure 2.**
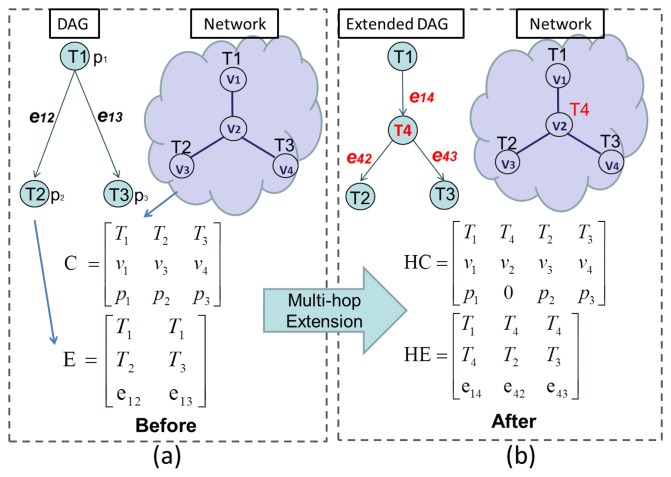
An example of the multi-hop extension process.

An example is illustrated in Figure 2a, which contains a mapping of a three-task DAG to a four-node network. The elements in the first row are the tasks, while the corresponding places in the second row and third row stand for node ID and computation load, respectively. By observing either the matrix C or the network, it can be seen that *T*_1_ is allocated to *v*_1_, and *T*_1_'s child tasks, *T*_2_ and *T*_3_, are allocated to *v*_3_ and *v*_4_, respectively. Figure 2a also demonstrates the communication relation amongst tasks, modeled by a three-by-*γ* matrix, *E*, called the *edge*, where *γ* is equal to the total number of edges in the DAG. The three elements in each column of *E* represent the sender task (*T*_1_), the receiver tasks (*T*_2_ or *T*_3_) and the total amount of data (*e*_12_ or *e*_23_) that need to be transmitted.

In order to consider multihop communication costs, *C* and *E* must be modified. First, relay nodes are determined by a routing algorithm (e.g., minimum-hop Dijkstra [27]), and then, *C* and *E* are extended by adding information on multi-hop relays. This process is called *multi-hop extension*, and the extended matrices, *C* and *E*, are named the *hyper-chromosome*(*HC*) and *hyper-edge*(*HE*). This is shown in Figure 2b. Here, task *T*_4_ is called a *routing task*, with no processing cost, and is allocated to the relay node, *v*_2_, which connects *v*_1_ to *v*_3_ and *v*_4_. This extension corresponds to the second column of *HC*. As a result, virtual links from *T*_1_ to *T*_4_, from *T*_4_ to *T*_2_ and from *T*_4_ to *T*_3_ are created, as shown in the extended DAG in Figure 2b. The second and third columns of *HE* correspond to these new links.

Based on *HC* and *HE*, the time and energy costs of both multi-hop communication and computation at the assigned nodes can be calculated, and the network lifetime, *NL*, and the schedule length, *SL*, required by the objective function can be obtained.

### 3.2. Computation of the Network Lifetime (NL)

In order to calculate the expected lifetime *NL*(*s*), the computational costs, *p_i_*, and the edge costs, *e_ij_*, first need to be converted into the actual time and energy costs at the assigned nodes, based on processing speeds and communication distances. *NL*(*s*) is calculated by:

(2) $$NL(s)=\min\{\frac{R^{v_{j}}}{E_{\text{total}}^{v_{j}}}|\;j=1,2,\cdots ,M\}$$

where *R^v^* denotes node *v*'s residual energy level and $E_{\text{total}}^{v}$ is the total energy consumption during one round of DAG execution at node *v*. *R^v^* can be obtained from periodic node reports whose signalling cost is explained in Section 4.5.

The total cost, $E_{\text{total}}^{v}$, includes the computation costs, $E_{p}^{v}$, of all data processing tasks in *HC* and the communication costs, $E_{t}^{v}$ and $E_{r}^{v}$, of data transfer tasks given in *HE*, as follows:

(3) $$E_{\text{total}}^{v}=\sum_{T\in HC}E_{p}^{v}(T)+\sum_{T\in HE}E_{t}^{v}(T)+\sum_{T\in HE}E_{r}^{v}(T)$$

The energy consumption of processing *T* on *v* is $E_{p}^{v}(T)=t_{T}^{v}P^{v}$, where *P^v^* is the power consumption of node *v*'s processor. $t_{T}^{v}$ is the processing time (sec) of task *T* at node *v*, calculated by $t_{T}^{v}=\frac{p_{T}}{f_{v}}$, where *p_T_* is the computational load (bits) of *T* and *f_v_* stands for *v*'s processor speed (bits/sec).

A popular short-range communication energy model [28] is used to calculate communication energy consumption costs:

$$\begin{array}{l} E_{t}^{v}=\{\begin{array}{cc} (be_{t}+{ɛ}_{fs}\cdot d^{2})\cdot e_{ij}, & \text{if}\;d<d_{0}\\ (be_{t}+{ɛ}_{mp}\cdot d^{4})\cdot e_{ij}, & \text{if}\;d\geq d_{0} \end{array}\\ E_{r}^{v}=be_{r}\cdot e_{ij} \end{array}$$

where the baseline energy consumption in operating the transmitter and receiver radios are expressed as *be_t_* and *be_r_*, respectively. The transmission energy consumption is denoted by either the ‘free space’ channel model (*ε_fs_d*^2^) or the ‘multi-path fading’ channel model (*ε_mp_d*^4^), depending on the distance, *d*, between the two nodes and a distance threshold, *d*_0_[28].

### 3.3. Computation of the Schedule Length (SL)

Based on *HC* and *HE*, multi-hop scheduling should provide a suitable schedule length, *SL*, that enables simultaneously occurring communication and parallel processing events. However, interference between different transmission events and the overlap of task execution at each node should be avoided. Therefore, the same scheduling method proposed in [13] is applied, where a two-hop interference model [29] is used and a medium access delay is introduced, such that the sender of a communication event does not cause interference on its one-hop receivers, and *vice versa*. Details of computation scheduling and communication tasks can be found in [13].

## 4. The DTAS Framework

**Figure 3.**
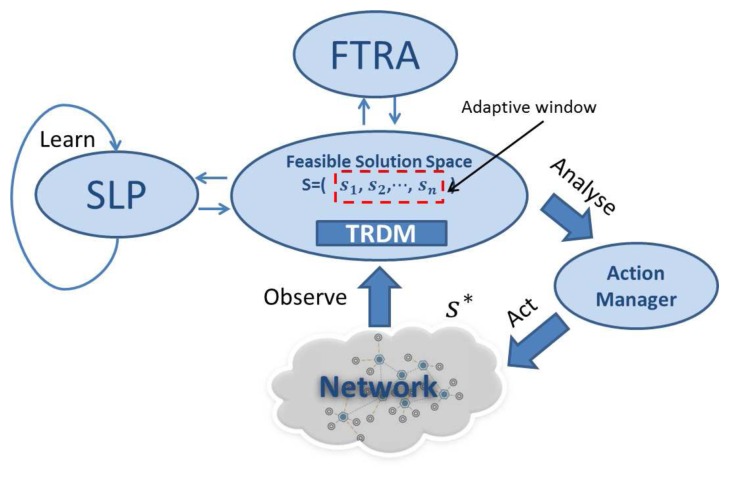
The dynamic task allocation framework.

Static task allocation in multi-hop wireless networks shown in the previous section is already a complex process and has been shown to be NP-hard (Non-deterministic Polynomial-time hard) [11], while network dynamics further complicates the problem. For instance, node mobility and failure events can easily render a task allocation solution invalid, in which case, a complete re-run of the task allocation algorithm from scratch is not a feasible option, since this is computationally inefficient. Therefore, a purely GA or sophisticated heuristics, which have to be re-run after each network update, are not suitable for dynamic MWSNs. On the other hand, an optimal initialization with a simple recovery process also struggles to solve this problem, as its performance reduces over time due to network topology changes. As a remedy to this problem, DTAS is proposed in this paper, which is designed to combine the strength of both heuristic (efficient) and GA (evolutionary) algorithms, to capture network dynamicity and to quickly re-adjust task allocation solutions to newly emerging conditions. DTAS is illustrated in Figure 3.

DTAS has the following three main components:

1. *Self-learning process (SLP)*: SLP is a periodically operated GA-based system component that runs in the system background and performs parallel optimization of task allocation solutions. Unlike conventional GAs, solutions at each evolutional stage of SLP can be modified based on changes in network topology. Hence, SLP results can be continuously updated and evolved.
2. *Fast Task Recovery Algorithm (FTRA)*: FTRA is a low-complexity event-triggered system component, which updates SLP solutions. FTRA can quickly perform task re-allocation when node or link failures occur.
3. *Task Re-allocation Decision Maker (TRDM)*: TRDM interacts with other system components and makes task re-allocation decisions based on different network conditions.

As seen in Figure 3, the gateway node maintains a *feasible solution space*, which contains the best set of solutions *S* = (*s*_1_, *s*_2_, ⋯, *s_n_*) that are suitable for the latest network conditions. *S* is an empirical data history that is used to train existing solutions in order to improve future system performance and it is periodically updated by SLP and has an adaptive window size of *n*, which virtually limits the time period necessary to renew *S. n* is modified based on network dynamics and the processing capability of the gateway device. Based on the current network conditions, TRDM picks the best available solution s* ∈ *S* and passes it to the *Action Manager*, which then performs task re-allocation in the network.

A node and a communication link that are assigned with tasks by s* are named as an *active node* (*v_a_*) and an *active link* (*l_a_*), respectively. In the event of an active node or link failure or multiple such failures, the execution of the current DAG round is stopped and an alternative best-fit solution in *S* can immediately be invoked by TRDM. However, if no valid solution is found in *S*, then TRDM asks FTRA to provide alternative solutions. Then, the new round of DAG task execution would restart, once the affected tasks have been allocation and rescheduled.

### 4.1. Solution Space Initialization

When an application arrives, the solution space, *S*, is first initialized and, then, dynamically updated by DTAS components. Multi-heuristic approaches are used in order to provide a suitable system start-up. The majority of the initial solution space, *S*, is generated by a *Minimum Hop Count (MHC)* algorithm (detailed in the next section), while the rest are provided by other simple heuristics, such as Random (Tasks are randomly allocated to nodes.) and Greedy (Tasks are assigned to a single, but relatively powerful, node in order to reduce communication costs). The complementary use of such a multi-heuristic scheme provides some level of diversity to the initial solution space, which prevents *S* from getting stuck in a local optimum.

### 4.2. The Minimum Hop Count Algorithm (MHC)

MHC is used for system initialization, as well as being implemented in the FTRA algorithm to reallocate tasks when network failure events occur. Since a fast system response is normally expected for these two processes, MHC is designed to assign tasks based on hop distance only, rather than calculating SL and NL. This is because hop distance directly affects communication costs, which normally dominate the total consumption (in both time and energy) [13,19]. Therefore, a hop distance-based fuzzy search can efficiently reduce algorithm execution time and provide quick sub-optimal solutions to the system. Details of MHC are provided below.

In a task graph, G, the set of tasks that precede a task, *T*, is denoted by *T_pre_*, and the set of nodes that *T_pre_* is assigned to is *V_pre_*. A task that does not have any predecessor tasks is called a *source task*. The assignment of source tasks may depend on individual applications. For instance, in wireless sensor networks, sensing tasks are source tasks and might be fixed at specific nodes. However, successor tasks are often processed at other nodes in the network. In such cases, MHC is used to find cost-effective allocations for the successor tasks. The pseudo-code of MHC is presented in Algorithm 1, which allocates a task, *T*, to a node, *Node(T)*.

In order to reduce the chance of high-cost multi-hop communication, the candidates for assigning task *T* are chosen among the nodes that have the minimum Total Hop Count (THC) to the nodes in *V_pre_*. This is performed at lines 9-13 of Algorithm 1, where hop counts $HC_{v_{i}}^{v_{j}}$ from *v_i_* to individual nodes *v_j_* ∈ *V_pre_* are summed to calculate *THC_i_* for *v_i_*. Then, those nodes *v_i_* with *THC_i_≤* min(*THC*) + *η* are selected as candidates, and the final node is randomly picked among these candidates. *η* is used to limit the number of candidate nodes.

**Figure 4.**
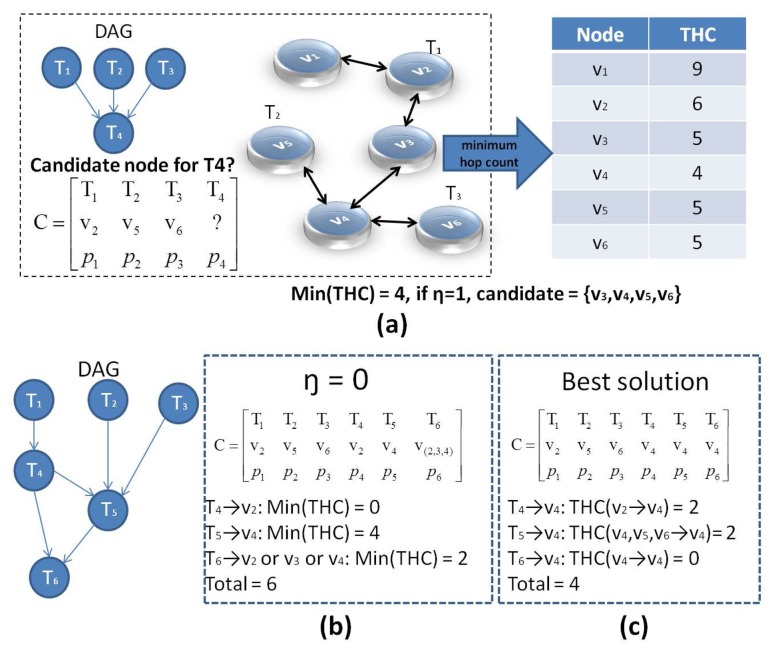
Minimum Hop Count candidate selection.

An example of the MHC algorithm is shown in Figure 4. *T*_1_, *T*_2_ and *T*_3_ have been assigned to *v*_2_, *v*_5_ and *v*_6_, respectively. Hence, the goal is to allocate *T*_4_ to a suitable node. If *T*_4_ is assigned to *v*_1_, the hop count (HC) from *T*_1_ to *T*_4_ is $HC_{v_{2}}^{v_{1}}=1$. Similarly, the HC from *T*_2_ and *T*_3_ to *T*_4_ can be obtained as $HC_{v_{5}}^{v_{1}}=4$ and $HC_{v_{6}}^{v_{1}}=4$, respectively. By summing the three HCs, we have *THC*(*v*_2_,*v*_5_,*v*_6_ → *v*_1_) = 9. The table in Figure 4a shows that assigning *T*_4_ to *v*_4_ provides the minimum *THC* among all nodes. When *η* = 1, the candidate set is {*v*_3_, *v*_4_, *v*_5_, *v*_6_}.



**Algorithm 1** The Minimum Hop Count (MHC) algorithm.
1:At node *v_i_*:2:**for** each *T* ∈ *G* based on a task scheduling sequence (TSS) **do**3: *candidates* ← Ø4: **if**
*T_pre_* = Ø **then**5:   Assign *T* as a source task to *v_i_*;6:  continue;7: **else**8:  Determine *V_pre_*9:  **for** each node *v_i_* ∈ *V*
**do**10:    *THC_i_* ← 0;11:   **for** each *v_j_* ∈ *V_pre_*
**do**12:    
$THC_{i}\leftarrow THC_{i}+HC_{v_{i}}^{v_{j}};$13:   **end for**14:  **end for**15: **end if**16: **for** each node *v_i_* ∈*V*
**do**17:  **if**
*THC_i_* ≤ min(*THC*) + *η*
**then**18:   *candidates* ← {*candidates*, *v_i_*};19:  **end if**20: **end for**21: % randomly select a node from *candidates*22: *Node*(*T*) = *rand*(*candidates*);23:**end for**


Note that, if *η* is set to zero, only nodes with the minimum *THC* can be selected as candidate. As a result, the solution space loses its robustness, and the final solution may not be the best possible one. Figure 4b shows another example to demonstrate this. The DAG now includes six tasks, where *T*_1_, *T*_2_ and *T*_3_ have been assigned to *v*_2_, *v*_5_ and *v*_6_, similar to the previous example in Figure 4a. When *η* = 0, *T*_4_, *T*_5_ and *T*_6_ can be assigned to different combinations of nodes, as shown in the matrices *C* of Figure 4b, c. In Figure 4b, *v*_2_ is directly chosen for *T*_4_ with *min*(*THC*) = 0, and then, *T*_5_ is allocated to *v*_4_ with *min*(*THC*) = 4. Finally, *T*_6_ can be assigned to either *v*_2_, *v*_3_ or *v*_4_ with the same *min*(*THC*) = 2, and the aggregate *THC* reaches six. In contrast, if *T*_4_, *T*_5_ and *T*_6_ are assigned to *v*_4_, as shown in Figure 4c, the aggregate *THC* becomes four, which is a better result. Therefore, in complicated scenarios with more tasks and various edge costs (*e_ij_*), *η* = 0 may not lead to the best possible solution. Thus, the purpose of the MHC algorithm is to eliminate inefficient or high-cost solutions and produce candidates that are more likely to become the best solution. A further refinement among these candidates to pick the best solution s* is performed by SLP, which is described in the next section.

### 4.3. The Self-Learning Process (SLP)

**Figure 5.**
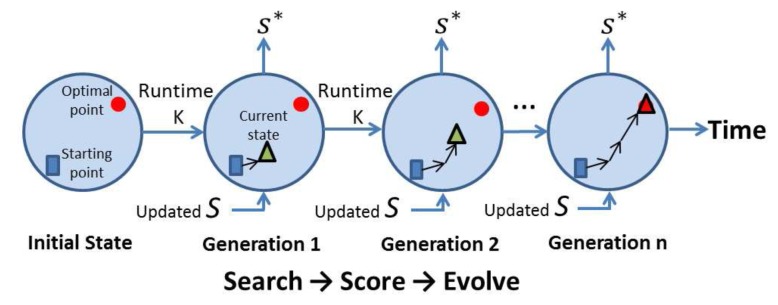
The self-learning process (SLP).

In a slowly changing environment, using past solutions as a benchmark point provides suitable algorithm initialization when seeking new solutions. Based on this fact, SLP is applied to refine the solution set provided by MHC. SLP is a daemon process that continuously evolves the solution space, *S*, in order to generate new task allocations in every time period, *K*, as depicted in Figure 5. To reduce the algorithm complexity and system response delay, only one *GA generation* [14] is performed in each iteration. Details of the SLP GA used in each stage of the SLP process are presented in Section 4.9.

### 4.4. The Fast Task Recovery Algorithm (FTRA)

When active node failure (*V_f_*), link failure (*L_f_*) or multiple simultaneous failure events take place, event-triggered reports (detailed in Section 4.5) containing information about those failure events and corresponding network topology changes are sent back to the gateway (Please note, not all node/link failure events would effect the current allocation s*, which are not belonging to *V_f_* and *V_l_*, e.g., a node fails, but with no tasks assigned.). The FTRA algorithm is then used to perform task re-allocations.

The FTRA algorithm is shown in Algorithm 2. When a node, *v_i_*, in *C* fails (line 3), its tasks have to be re-allocated. If any *T* ∈ *T_defect_* is a source task (line 8), then FTRA randomly assigns this task to one of the neighbour nodes. Otherwise, the MHC algorithm (line 13) is used to choose the replacement node. Then, multi-hop extension is performed (line 21) in order to avoid any resulting broken links.



**Algorithm 2** The Fast Task Recovery Algorithm (FTRA) algorithm.
**Require:**
*C*, *E*, *V_f_***Ensure:** New *HC*, *HE*1:% Detect the set of defected tasks *T_defect_*2:**for** each node *v_i_* ∈ *C*
**do**3: **if**
*v_i_* ∈*v_f_*
**then**4:  Include all *T* assigned on *v_i_* in *T_defect_*5:  % Fix node failure6:  **for** each *T* ∈*T_defect_*
**do**7:   Find all *V_pre_* for *T*8:   **if**
*V_pre_* = Ø **then**9:    % Re-allocate source tasks10:    *n_i_* ← *v_i_*'s *one-hop neighbours*11:    % Randomly select a node from *n_i_*12:    *Node*(*T*) = *rand*(*n_i_*)13:   **else**14:    *Node*(*T*) = *MHC*(*v_i_*)15:   **end if**16:   Update *C* with *T* and *Node*(*T*)17:  **end for**18: **end if**19:**end for**20:% Fix possible link failure: perform multi-hop extension %21:*C* =⇒HC, *E* =⇒HE


### 4.5. The Task Reallocation Decision Maker (TRDM)

**Figure 6.**
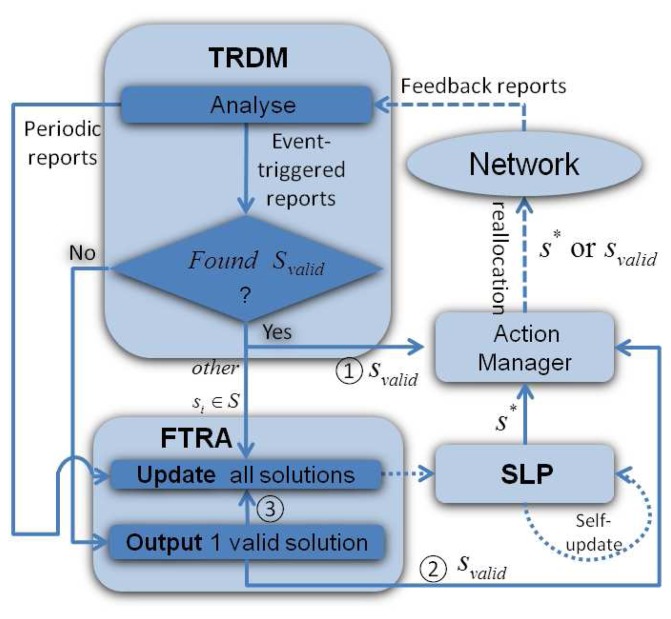
Task Reallocation Decision Maker (TRDM) function flowchart.

TRDM is the decision maker and the central component of DTAS, which realizes seamless collaboration and interaction between FTRA and SLP, as seen in Figure 6. It makes decisions according to feedback from the network: (1) periodic reports (no topology change) and (2) event-triggered reports (active node failure events).

#### 1. Periodic reports

Each node in DTAS periodically sends a *REPORT* message to the gateway, providing its latest set of neighbours and residual energy level. Based on this information, the gateway updates its knowledge of the network topology and can re-calculate *NL* by Equation (1), so that the latest energy distribution is taken into account when new task allocation solutions are generated. The frequency of periodic reports is equal to the algorithm runtime, *K*, as too frequent reports cause additional signalling costs, while a long report period may have a poor adaptation to the network dynamicity. Furthermore, if the TRDM misses a periodic report, it basically assumes that it would receive the next one and do nothing. However, in the unlikely case that if the TRDM have not received any periodic report from a particular node for a long time, including event-triggered reports initiated by its neighbours, it reports the failure of that node. Then, the TRDM may send additional report requests, which come with the cost of more contro overhead.

Upon receiving all node reports, TRDM asks FTRA to examine all existing solutions *s* ∈ *S*. Periodic reports do not include situations in which *V_f_* affects s*, since such cases are handled by event-triggered reports. However, changes in network topology and residual node energy levels may influence other existing solutions in *S*. FTRA identifies any such affected solutions and makes task re-allocation accordingly.

Once FTRA completes its modifications on *S*, TRDM initiates SLP. At the end of SLP, *S* may contain a better solution, *s_new_*, than the current one, s*, in which case, TRDM selects the new solution s* = *s_new_* and passes it to the Action Manager for a task re-allocation. The gateway broadcasts an *ALLOCATION* message, which delivers the new task assignments to the nodes in s* and releases the nodes that currently hold these task allocations.

#### 2. Event-triggered reports

An event-triggered report is generated when an active node/link failure occurs (*V_f_*/*L_f_* ≠ Ø) by one of the neighbouring nodes. In this case, the current solution s* is directly affected, and hence, an urgent task re-allocation is required. The event-triggered reports have a high priority and are continuously to be sent, until they reach the TRDM. Upon receiving this failure notification, the gateway broadcasts a *REPORT_REQUEST* message, asking for the latest residual energy levels and neighbour lists. Then, each node sends a *REPORT* message to the gateway.

The first step that TRDM takes is to search *S* for any solution that fits the current network conditions (see step ➀ in Figure 6). This may help the system quickly recover from the failure event. Basically, the best-fit solution, *s_valid_*, is chosen based on a ranking table that records each solution's performance profile. s* is usually the one listed at the top of the ranking table. Hence, *s_valid_* can be determined by choosing the second best one in the ranking table, which is not affected by *V_f_*. Then, *s_valid_* is passed to the Action Manager for immediate task re-allocation. Details of the ranking table are provided in Section 4.9.

If there is no solution *s_valid_* that fits the new network conditions, TRDM consults FTRA, which then provides a valid solution to the Action Manager (see ➁ in Figure 6). The rest of the solution space is also examined and updated by FTRA (Figure 6 ➂), although not provided as an output to the Action Manager. This is a measure towards adapting *S* according to the knowledge of the latest conditions acquired via the event-triggered report.

### 4.6. DTAS Solution Selection and Evolution

In this section, the generation of each solution *s* with multiple objectives is briefly described, and then, the GA used in SLP is presented in detail.

### 4.7. A Hybrid Fitness Function

In GAs, a solution is ranked by a fitness value that represents how suitable the solution is to meet design objectives. A solution is more desirable if it has a high fitness value. In DTAS, the two parameters, NL and SL, are used to compute a single “hybrid” fitness value for a task allocation solutions *s* as follows:

(4) $$\begin{array}{l} \text{fitness}(s)=\frac{NL(s)}{\max(NL(S))}-\alpha \frac{SL(s)}{\max(SL(S))}\\ \alpha =\{\begin{array}{ll} 0\;, & SL(s)\leq t_{\text{deadline}}\\ \frac{\min(NL(S))}{\max(NL(S))}\;, & SL(s)\leq t_{\text{deadline}} \end{array} \end{array}$$

where a candidate solution's network lifetime, *NL*(*s*), and schedule length, *SL*(*s*), are normalized by the corresponding maximum values in the solution space, *S*. The rationale behind this normalization is to capture the relative significance of *s* among all solutions in *S*. Here, *α* is a tuning parameter that provides a weight between the two fitness parameters. We have *α* = 0 when the schedule length meets the deadline. A non-zero value of *α* lowers the fitness value depending on how large *SL*(*s*) is, which is a measure that penalizes those solutions with a large *SL*. Note that Equation (4) favours the solutions with a larger *NL*.

Since a fitness value in SLP GA has to be a non-negative value (applying the Roulette-Wheel selection scheme [14]), *fitness*(*s*) ≥ 0, hence:

(5) $$\alpha \leq \frac{NL(s)}{\max(NL(S))}\times \frac{\max(SL(S))}{SL(s)}$$

In order to guarantee that *fitness*(*s*) ≥ 0, ∀*s* ∈ *S*, we set *SL*(*s*) = *max*(*SL*(*S*)) and *NL*(*s*) = *min* (*NL*(*S*)). This provides the lower bound for *α* in Equation (5), which is $\alpha =\frac{\max(NL(S))}{\max(NL(S))}$.

Using the hybrid fitness value, *fitness*(*s*), *NL*(*s*) and *SL*(*s*) are calculated, and the solutions are sorted and indexed in the ranking table.

### 4.8. Adaptive Window Size n

In DTAS, an adaptive window size *n* is defined to adjust the size of the solution space, which essentially controls the trade-off between complexity and performance. A larger value of *n* increases the algorithm runtime, K, but has a higher probability of offering better solutions to meet the design objectives. In contrast, a small value of *n* provides a short algorithm response time with results of lower quality, which may still be suitable for frequently changing networks.

### 4.9. The SLP GA

**Figure 7.**
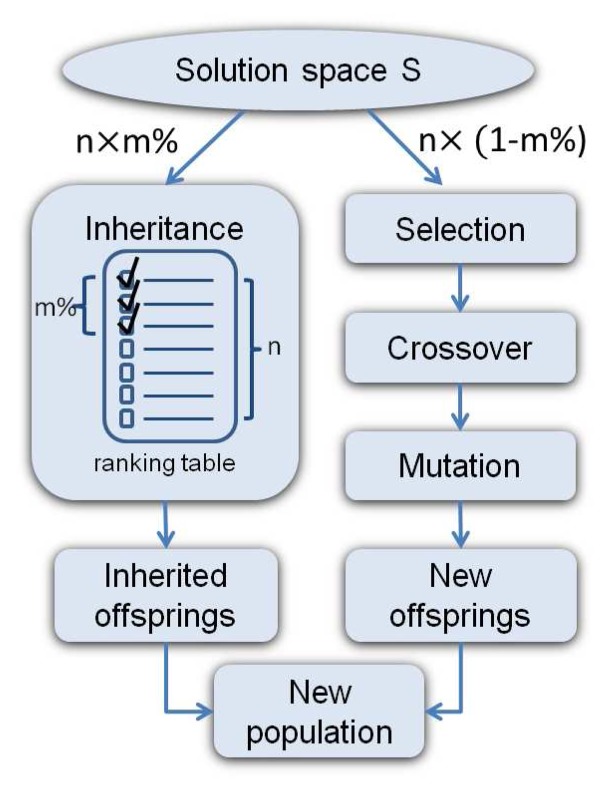
The SLP genetic algorithm (GA).

Conventional GAs normally terminate and produce results after running for several iterations or the optimal solution has been identified. In contrast, the SLP GA outputs and uses temporarily sub-optimal solutions in each SLP run, and the top ranked solution in the ranking table is selected as s*. SLP GA also stops under the satisfaction of two conditions: i) No event-triggered report has been received. This means SLP can always work on a stable solution space. ii) No better solution is found by the SLP GA before a pre-defined timer expires, which is set to *x · K* rounds. Nonetheless, once FTRA is used, the timer will set to its default value. Typical GA operations are employed in SLP GA as shown in Figure 7, where each GA step is briefly described below.

#### Inheritance

In order to keep the good allocations of the current solution space S, the top m% of n chromosomes in the ranking table are inherited to the next generation, while the rest of the *n* ×(1− *m*%) chromosomes are produced via the *selection*, *crossover* and *mutation* process described below.

The ranking table is initially sorted in a decreasing order of the combined fitness value (*fitness*). After that, chromosomes that can meet the deadline requirement are moved to the top of the table. In this way, chromosomes that satisfy the application deadline while having larger fitness values are placed in the upper rows of the ranking table. In case none of them in the current population can meet the application deadline, the ranking table is re-sorted in an increasing order of *SL*, such that the chromosomes with a shorter schedule length can be inherited.

#### Selection

The selection process chooses the most suitable chromosomes to crossover, which produces new offspring. In SLP GA, the well-known Roulette-Wheel scheme [14] is used, where a chromosome with a better fitness value has a higher probability of being selected.

#### Crossover

**Figure 8.**
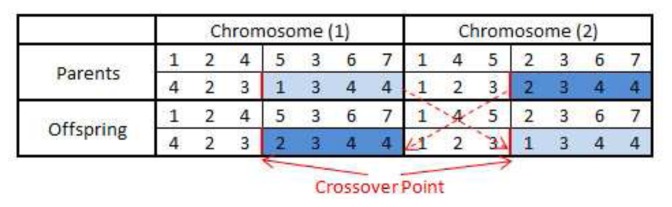
Example of crossover.

The crossover operation is performed on each selected chromosome pair and produces new chromosomes by recombination of some portions of both parent's genetic materials (task allocations). In order to keep the topological execution order of the DAG, the tasks in the first row of the chromosome matrix C remain unchanged, while the mapped nodes in the second row are swapped after the crossover point. An example of single point crossover is shown in Figure 8 where the mapped nodes in the second row are switched over after the crossover point. In this way, the purpose of the crossover has been achieved, and the execution sequence of tasks in the DAG is still preserved.

Furthermore, crossover only applies to the original chromosome rather than *HC*, due to the exclusive routing task mapping. Therefore, new *HC* and *HE* need to be regenerated for the offspring in order to calculate their fitness values. Please note that the crossover may or may not produce better offspring than their parents. However, if both parents have good ‘genes’, there is a higher probability of producing better survival chromosomes.

#### Mutation

In order to maintain genetic diversity and reduce the probability of the solution that GA produces a local maximum, the mutation process avoids having too similar chromosomes. Two types of mutation are employed: one is on a ‘task allocation’ basis (each chromosome has a probability of *ϕ* to change a randomly selected tasking mapping to another node); the other is on a ‘chromosome’ basis (each chromosome has a probability of *ϕ* being completely replaced by a randomly created new chromosome), where *ϕ* is the mutation rate.

### 4.10. Complexity Analysis

Given a DAG with N tasks and a network with M nodes, the complexity of the fitness function (calculation of *NL* and *SL)* is 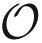
(*N* · *ε*), where 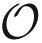
(*ε*) is the complexity of the routing algorithm (e.g., if Dijkstra [27], 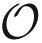
(*ε*) = 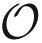
(*M*^2^)). Therefore, DTAS has an algorithm complexity of 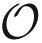
(*N* · *ε* · *n*) for SLP, where the adaptive window size *n* denotes the number of the solutions that are evaluated for each SLP stage. The algorithm complexities of selected competitors are shown in Table 1, where *e* is the number of edges in DAG, *x* represents the chromosome number in GA population and *y* is the generation number.

Please note that the algorithm complexity determines how often each algorithm can update its solution; hence, it directly affects the system's adaptability to network dynamics. Since *n* ≪ *e* · *M* and *n* ⩽ *x*, SLP in DTAS shows less complexity compared to MTMS and ITAS. Greedy has the least algorithm complexity compared to the others, as seen in Table 1, yet it delivers low quality results that hinder performance, as presented in Section 5. Numerical results of each algorithm's runtime, performance and their adaptability to network dynamics are shown in Section 5.3.

## 5. Results

The DTAS framework is evaluated through simulations. To the best of our knowledge, this is the first study to address such a complex DAG-based task allocation problem in a multi-hop and mobile environment. Hence, two classic heuristic algorithms and a conventional GA-based algorithm are picked as benchmark competitors:

***Greedy*** [16]: The Greedy algorithm assigns most of the tasks to a powerful node, e.g., the gateway. Hence, raw data need to be first transmitted to the gateway and then processed there. Greedy can be quickly re-run to perform a task re-allocation once network changes occur.
***MTMS*** [11]: MTMS is a well-known cross-layer task allocation algorithm for multi-hop wireless networks. It performs multi-objective optimization, which aims at minimizing the total energy consumption while meeting the user deadline.
***ITAS*** [13]: ITAS is a conventional GA-based multi-objective optimization algorithm that also performs complex task allocation in multi-hop wireless networks.

### 5.1. Simulation Setup

#### 5.1.1. Application DAG Generation

The parameters to generate a random DAG are obtained from MTMS [11]. For a single object tracking case, 256 × 256 images have an average computation load of approximately 300 KCC (kilo-clock-cycle) for the tasks, and we assume 800 bits of communication data that need to be transmitted between the tasks. Then, each communication and computation workload of the DAG tasks are generated with a standard deviation of 25% of the above average values.

#### 5.1.2. Network

The network consists of two node types: super nodes (10-20%) and normal nodes. A normal node has a processor speed of 133 *MHz* (e.g., an Intel Strong Arm 1100 processor with 150 *MIPS* [19]). The power consumption for such a processor is *Pc* = 200 *mW*, and each node has a battery energy of 2,000 *J* (2 × AAA NiCad batteries). On the other hand, super nodes have a 206 *MHz* processing speed with 235 *MIPS*, *Pc* = 400 *mW*, and a battery energy of 4, 000 *J*. The communication bandwidth is 250 *Kbps*, and the communication range for all nodes is 30 *m* on the ISM Bands (industrial, scientific and medica bands). Based on those parameter settings, the average time cost to process a task and transmit information (single-hop) between them are around 2.26 ms and 3.2 ms, respectively. Thus, in simulations, the application deadline varies between [20, 30, ⋯ 80] ms(by default 40 *ms)* considering parallel processing and multi-hop communications, which we believe are reasonable values for such applications based on the number of tasks and the number of nodes we used. In addition, two types of gateway (GW) devices with different processing capabilities are considered: GW-A (e.g., a PC or laptop) with a 2 *GHz* processor and GW-B (e.g., a smart phone) with a 1 *GHz* processor. Unless specified otherwise, GW-A is used as the default gateway type in performance evaluations. The gateway is fixed at the centre of a 100 × 100 *m*^2^ network area, while the other nodes have an equal movement probability *p_move_* with a moving speed of *ν_move_*. In all simulations, the GA parameters are pre-optimized based on [13], and *η* = 1 is chosen for the MHC algorithm. The average overhead packet length of the periodic and event-driven reports is assumed to be 200 bits.

In the following, results of independent simulations are presented, by altering a single simulation parameter each time, so that any changes in performance would be based solely on this parameter. All results are averages of more than 400 simulation runs.

### 5.2. Effect of Node Mobility on Network Dynamics

**Figure 9.**
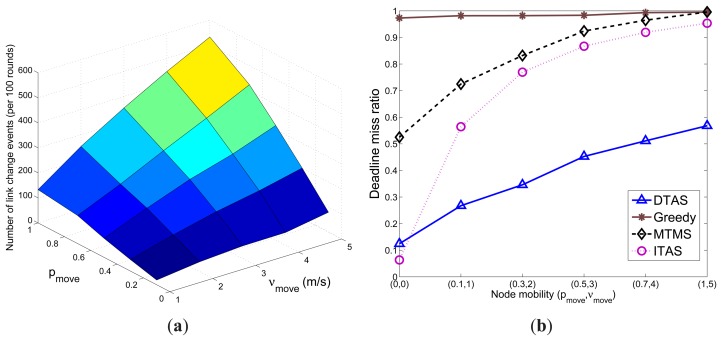
Impact of *p_move_* and *ν_move_*. (**a**) Network link-change events; (**b**) Deadline miss ratio.

In this section, first the effect of *p_move_* and *ν_move_* on network dynamicity (represented by the number of link-change event) are shown. Here, a link-change event is either a link breakage event when nodes move out of each other's communication range, or the formation of a new link as a result of node mobility. Results in Figure 9a demonstrate that the total number of link-change events occur more frequently with a larger probability *p_move_* of node mobility (more nodes move) and/or a higher node speed *ν_move_*. The performances of the algorithms in meeting the task execution deadline degrade when node mobility gets higher, which can be observed in Figure 9b. ITAS and MTMS have a higher performance degradation, due to their algorithm complexity. Greedy's performance is quite stable, as it assigns most of the tasks on a single node; thus, it is less affected by topology changes. On the other hand, it has the highest deadline miss ratio, due to the ‘hotspot’ problem. Although DTAS shows the best performance compared to MTMS, ITAS and Greedy, it is inevitable that the deadline miss ratio of DTAS also increases significantly when more link-change events take place. Nevertheless, DTAS shows the best performance under the tested mobile environment. Further simulation results on adaptability to network dynamics can be found in Section 5.3.

In the rest of this section, *ν_move_* is randomly chosen between [1, 2] m/s in the following tests as a typical pedestrian speed. Different *p_move_* values are used to represent different levels of network dynamicity.

### 5.3. Algorithm Adaptability to Network Dynamics

The goal of this set of simulations is: (1) To compare the adaptation of the DTAS to network dynamics compared to Greedy, MTMS and ITAS; (2) To test each algorithm's performance in meeting the design objectives.

Table 2 illustrates the runtime of each algorithm. Obviously, the longer an algorithm takes to run and produce its solution, the lower the frequency that the algorithm can update its task allocation solution (s*). In order to observe this trade-off, another time unit is introduced to count the algorithm runtime, called the *Task Reallocation Frequency (TRF)*, shown in the last column of Table 2. TRF represents how often, in terms of application rounds, an algorithm can perform task re-allocation based on its algorithm runtime, where an *application round* is the basic time unit in simulations representing the completion time of the DAG.

In Table 2, it can be observed that Greedy is a fast algorithm, which is able to perform task re-allocation in every round. Thus, Greedy is re-run at each time when an urgent task re-allocation is required. In contrast, MTMS and conventional GA-based ITAS require a longer time to execute, due to their complex search mechanisms.

In order to evaluate the algorithm's adaptability to network dynamics, two test parameters are defined: *Expected System Performance* (ESP) and *Actual System Performance* (ASP). ESP is calculated by averaging the snapshots of TRF cycles, while ASP is averaged from samples collected at each round. To explain it in a simpler way, the ESP value can be treated as an algorithm's performance in static network conditions, while the ASP value shows how the algorithm actually performs under network dynamics. Therefore, if an algorithm is fast enough to perform task re-allocation in each round, then *ASP* = *ESP*; otherwise, the value of ASP may degrade over time. A large gap between the two values indicates poor adaptation to network dynamics.

**Figure 10.**
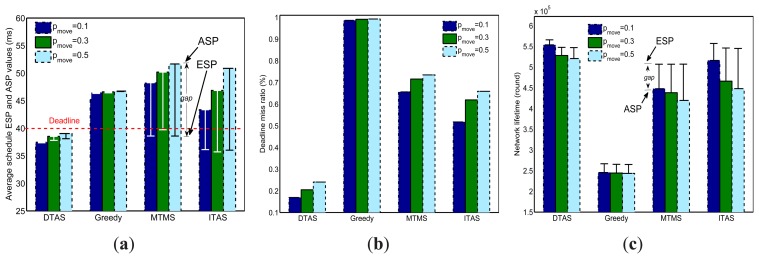
Comparison of algorithm adaptability to network dynamics. (**a**) Schedule length, Expected System Performance (ESP) *vs.* Actual System Performance (ASP); (**b**) Deadline miss ratio, ASP; (**c**) Network lifetime, ESP *vs.* ASP.

It can be observed in Figure 10a that the ESPs of the schedule lengths of MTMS, ITAS and DTAS are below the user deadline. However, the ASP of both MTMS and ITAS goes far beyond the deadline. In addition, when *p_move_* increases and more nodes are mobile, MTMS and ITAS have a larger gap between their corresponding ASP and ESP, due to their poor adaptability to network dynamics. Furthermore, as Greedy can simply be re-run when network dynamics occur, the ASP of Greedy is very close to its ESP. However, Greedy still cannot meet the deadline constraint, since it aggregates tasks to a single node, which becomes a processing bottleneck. In Figure 10b, significant performance improvement can be noticed for DTAS, which has a much lower ratio of missing the application deadline for all three node mobility cases compared with the other algorithms.

Figure 10c illustrates the comparison of results for network lifetime. As opposed to Greedy, the other algorithms distribute the total workload among more nodes. Therefore, a longer network lifetime can be noticed for MTMS, ITAS and DTAS. Furthermore, DTAS has a relatively smaller gap between the ASP and ESP of lifetime, and it can provide the longest lifetime values under the tested mobile environment.

### 5.4. Contribution of SLP

The system updater SLP is the unique feature of DTAS compared with the other algorithms (e.g., heuristic approaches), and it can work independently from other DTAS components. Hence, in this section, the contribution of SLP to the overall performance is evaluated.

Since the main objective of the fitness function in SLP is to meet the deadline constraint, system performances of the deadline miss ratio with or without the inclusion of SLP are shown in Table 3. A significant improvement in reducing the ratio of deadline misses can be observed when the system is equipped with SLP, with larger gains obtained for lower mobility cases.

### 5.5. Effect of Changing the Deadline Constraint

In this section, the DTAS's performance for different application deadlines is studied. DTAS is compared with only the best case scenarios of Greedy, MTMS and ITAS under different node mobility cases.

**Figure 11.**
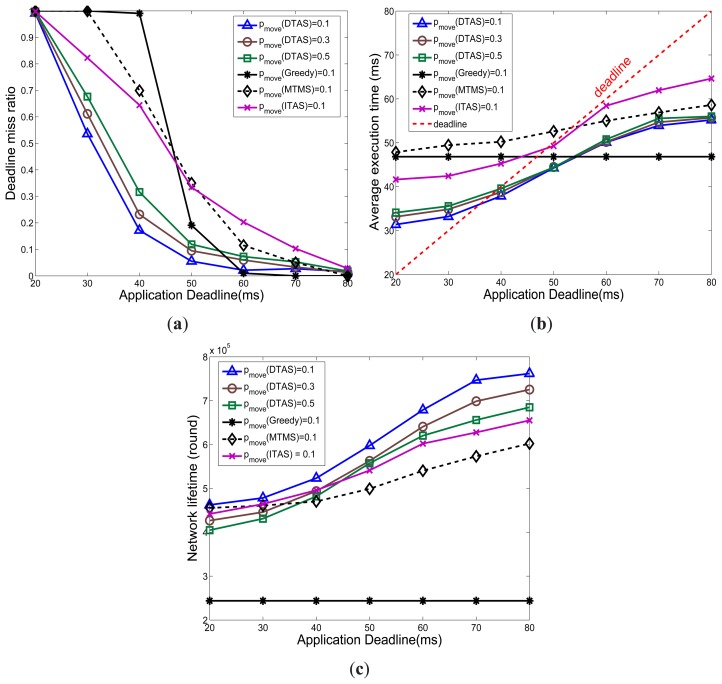
Effect of altering the deadline constraint. (**a**) Deadline miss ratio; (**b**) Average schedule length (SL); (**c**) Average Network lifetime (NL).

Results in Figure 11 demonstrate that DTAS is more adaptive to the deadline constraint and provides the lowest deadline miss ratio and the largest network lifetime. In fact, ITAS, MTMS and DTAS all promote resource sharing among nodes, which helps avoid network hotspots, yet DTAS shows a better performance, since MTMS and ITAS have poor adaptation to network dynamics. In contrast, a notably short network lifetime of Greedy can be noticed in Figure 11c, which stems from the fact that Greedy has an imbalanced task assignment that easily overloads some nodes, creating traffic or processing hot-spots. Greedy's performance is quite stable, as it does not consider the application deadline while making task allocation decisions.

### 5.6. Effect of Changing the Number of Nodes

**Figure 12.**
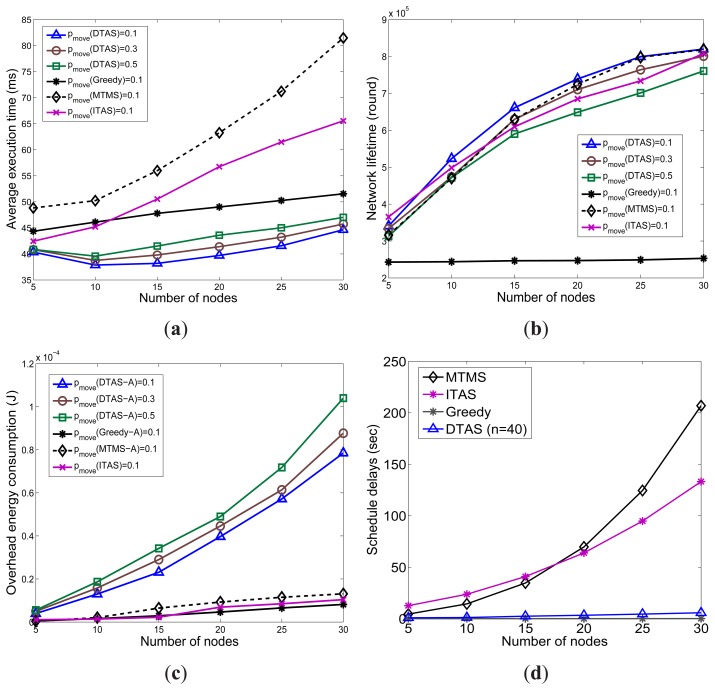
The effect of the number of nodes. (**a**) Average SL; (**b**) Average NL; (**c**) Energy consumption on overhead; (**d**) Scheduling delays.

In this section, the scalability of DTAS to networks with different numbers of nodes is studied.In Figure 12a, it can be observed that the average schedule length of DTAS first decreases when the number of nodes rises and, then, increases as more nodes join the network. This is because there is less chance to perform parallel processing when there are only a few nodes in the network. In addition, tasks are queued in node memory, which reduces processing efficiency. Thus, as more nodes are involved, the performance of DTAS in meeting task deadlines improves. However, when the network further expands, the search space increases exponentially, and finding a suitable solution is more difficult, resulting in a higher deadline miss ratio. Nevertheless, thanks to SLP and the adaptive window, DTAS still has the shortest average execution time and the lowest deadline miss ratio, as seen in Figure 12a.

On the other hand, the scalability of MTMS and ITAS is poor, due to their time complexity, as illustrated in Figure 12d. Furthermore, since Greedy gathers tasks on a few nodes, the increase in the number of nodes does not have much effect on the performance of Greedy, as shown in Figure 12b. When the number of nodes increases, the numbers of periodic and event-driven reports described in Section 4.5 increase dramatically. Therefore, the energy consumption on control overhead of DTAS also increases, as seen in Figure 12c. Although DTAS has a higher energy consumption stemming from its control overhead, it provides better and more balanced task allocation solutions to the network. Therefore, DTAS still has a marginal lifetime improvement (Figure 12b, with *p_move_* = 0.1) compared to ITAS and MTMS. Furthermore, since the schedule length has a higher priority in the objective function of DTAS, especially when a tight application deadline is imposed, DTAS mainly focuses on meeting the deadline rather than improving network lifetime. Nevertheless, DTAS has a much better lifetime improvement compared with ITAS and MTMS, as shown in Figure 11.

### 5.7. Effect of CCR

**Figure 13.**
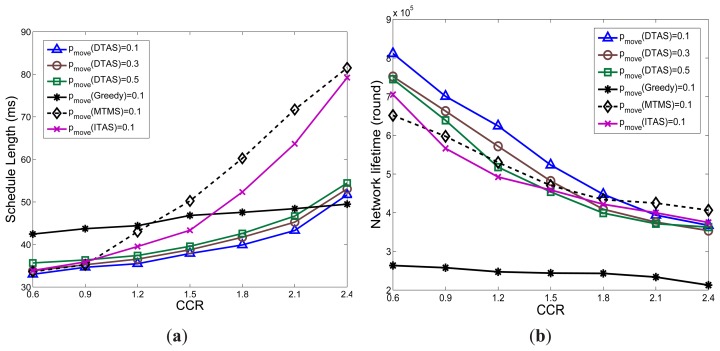
The effect of communication load to computation load ratio (CCR). (**a**) Average SL; (**b**) Average NL.

The communication load to computation load ratio (CCR) is an important parameter for DAG, as it indicates the ratio of the average energy consumption of the communication events to that of the computation activities. A larger value of CCR indicates that communication events dominate the total cost. When CCR increases, it incurs additional communication delays. Both MTMS and ITAS show a poor capability to avoid such communication delays, due to their algorithm complexity. The impact of this complexity on the SLs of MTMS and ITAS can be observed in Figure 13a. On the other hand, Greedy shows much less performance degradation and even outperforms DTAS when a larger value of CCR is employed, as demonstrated in Figure 13a. This is based on the fact that Greedy gathers most of the tasks on the same node, which reduces the communication cost. Nevertheless, due to the hot-spot problem, Greedy always shows the shortest network lifetime, as seen in Figure 13b. In addition, the larger the CCR value, the more difficult it is to meet the deadline. Hence, when CCR increases, DTAS spends most of its effort on reducing the schedule length rather than extending network lifetime. Thus, DTAS shows similar network lifetime degradation as MTMS and ITAS (Figure 13b), yet still provides the most balanced solution.

### 5.8. Effect of the Node Failure Probability

**Figure 14.**
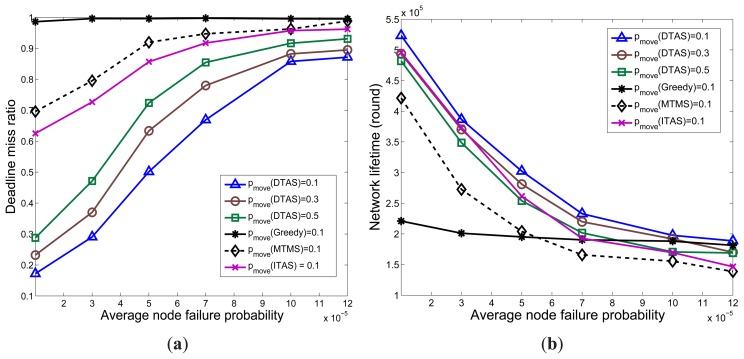
The effect of the average node failure probability (λ). (**a**) Deadline miss ratio; (**b**) Average NL.

The average node failure probability λ is varied in this section, and the results are shown in Figure 14. When λ increases, nodes are more likely to fail. Hence, all algorithms show poorer performance, yet DTAS performs better than Greedy, MTMS and ITAS, because the proposed FTRA algorithm can update the solution space once a node failure event happens. Since Greedy uses fewer nodes for task allocation compared to the other algorithms, it is less affected when the node failure probability rises. Nevertheless, Greedy cannot meet an arbitrary deadline and provides the shortest network lifetime, due to the hot-spot problem.

### 5.9. Selection of the Adaptive Window Size n

**Figure 15.**
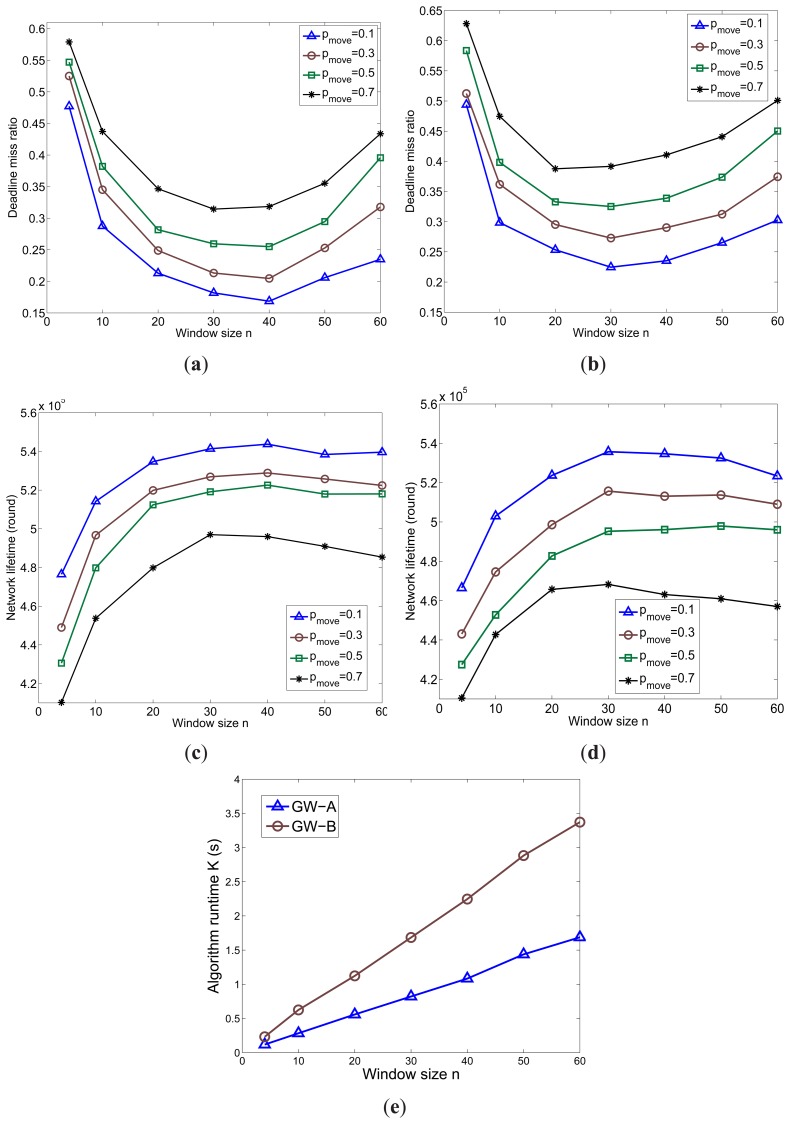
The effect of *n* and *p_move_* (*ν_move_* = 1 *m*/*s*). (**a**) Deadline miss ratio (GW-A); (**b**) Deadline miss ratio (GW-B); (**c**) Network lifetime (GW-A); (**d**) Network lifetime (GW-B); (**e**) Algorithm runtime.

**Figure 16.**
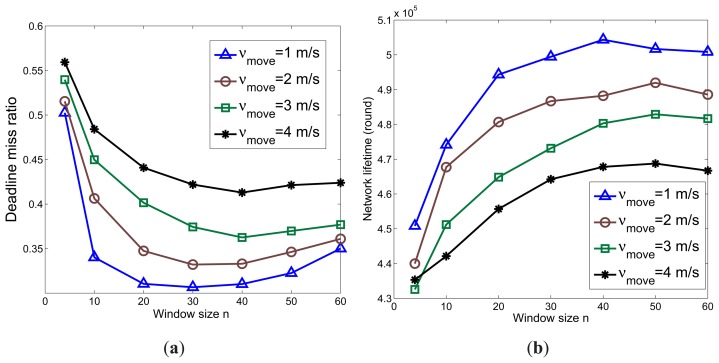
The effect of *n* and *ν_move_* (*p_move_* = 0.3). (**a**) Deadline miss ratio (GW-B); (**b**) Network lifetime (GW-B).

The impact of the window size on DTAS's performance in minimizing the deadline miss ratio and extending the network lifetime when tested on different gateways is illustrated in Figure 15. A more powerful gateway obviously can process a larger solution space for the same time period. Therefore, the performance of DTAS is better in GW-A than in GW-B. In addition, it can be clearly observed that the performance of DTAS first increases with more chromosomes joining the GA evolution process and, then, decreases when the window size becomes larger than a certain value. This is due to the fact that the larger window size *n* is, the longer it takes for DTAS to update its task allocation solution, as shown in Figure 15e. Hence, the solution adaptation to network dynamics degrades when *n* further increases. Therefore, a smaller window size is preferred for networks with higher node mobility. The best values of window size *n* (the lowest point in the deadline miss ratio curve) for GW-A and GW-B are 40 and 30, respectively, as observed in Figure 15a, b. A similar effect for different *ν_move_* values can be observed in Figure 16.

### 5.10. Effect of High Node Mobility

In this section, real-time performance curves with two distinguishing node mobility settings are provided for Greedy, MTMS and DTAS. Since the performance of ITAS is quite similar to MTMS in the high mobility case. Thus, it is not displayed for clear presentation purposes.

**Figure 17.**
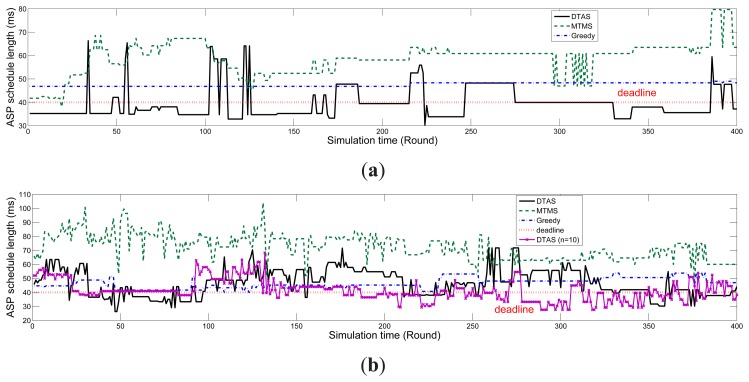
Comparison of algorithm SL for different node mobilities in real-time. (**a**) low mobility, *p_move_* = 0.3, *ν_move_* = 1 *m*/*s*; (**b**) high mobility, *p_move_* = 1, *ν_move_* = 5 *m*/*s*.

In the low mobility case, as demonstrated in Figure 17a, the DTAS curve is higher than the application deadline only momentarily a few times, whereas Greedy and MTMS consistently exceed the deadline. However, in the high mobility case, the DTAS curve frequently crosses the deadline curve, as seen in Figure 17b. This is due to the fact that SLP does not have sufficient time to evolve the solution space between two consecutive network change events. Therefore, decreasing the window size *n* or using a more powerful gateway can improve SLP's adaptability to mobility. In Figure 17b, such improvement can be observed when we have *n* = 10 for DTAS. Nonetheless, low mobility is our main targeting scenario, as mentioned before, where DTAS can show all its advantages.

## 6. Conclusion

In this paper, the DTAS framework is proposed for multi-hop multimedia wireless sensor networks with low mobility nodes, which can minimize the deadline miss ratio while also preserving and balancing node energy levels to extend network lifetime. This task allocation problem is very challenging when network dynamic and multi-hop wireless communication aspects are addressed simultaneously. A fast, but simple, heuristic algorithm, like Greedy, may only provide sub-optimal solutions. On the other hand, a sophisticated heuristic search algorithm, like MTMS, or a conventional GA-based solution, such as ITAS, performs relatively well under static network conditions, but has poor adaption to network dynamics, due to algorithm time-complexity. An integration of such a stage GA-based evolutional algorithm with an efficient fast heuristic running in between to adjust and correct the GA population is shown to be suitable for solving such complex and dynamic task allocation problems under a slowly changing environment. Furthermore, DTAS is able to make trade-offs between algorithm runtime and performance. Adaptive solutions can be produced according to how fast network changes occur, while also considering the processing capability of a controller device that needs to deal with such changes.

## Figures and Tables

**Table 1.** Algorithm complexity comparison.

**Algorithm**	**Complexity**
SLP	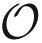 (*N* · *ε* · *n*)
Greedy [16]	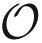 (*N*)
MTMS [11]	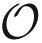 (*N* · *e* · *ε* · *M*)
ITAS [13]	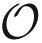 (*N* · *ε* · *x* · *y*)

**Table 2.** Algorithm runtime comparison. GW, gateway; DTAS, Dynamic Task Allocation and Scheduling; TRF, Task Reallocation Frequency.

**GW Hardware**	**Algorithm**	**Runtime K (s)**	**TRF (Rounds)**
GW-A (2 GHz)	DTAS (n = 40)	1.083	28
	Greedy	0.022	1
	MTMS	26.835	670
	ITAS	32.786	820

**Table 3.** Performance of SLP: deadline miss ratio.

**Algorithm**	*p_move_***= 0.1**	*p_move_***= 0.3**	*p_move_***= 0.5**
With SLP	0.17	0.23	0.32
Without SLP	0.40	0.42	0.44
